# Mr. Ring in Answer to Dr. Kinglake

**Published:** 1817-01

**Authors:** 


THE LONDON

### Medical and Physical Journal.

1 OF VOL. XXXVII.] JANUARY, 1817. [NO. 215.

"For many fortunate discoveries in medicine, and for the detection of numerous errors, the world is indebted to the rapid circulation of Monthly Journals; and there never existed any work to which the Faculty in EUROPE and AMERICA were under deeper obligations than to the Medical and Physical Journal of London, now forming a long, but an invaluable, series."—RUSH.

*For the London Medical and Physical Journal.*

### Mr. RING *in Answer to Dr. Kinglake.*

IN your Journal for November, I am represented as having brought against Dr. Kinglake the charge of fraudulently assuming a title; to this charge I plead Not guilty. Having been informed, and, as I then had reason to believe, by good authority, that Dr. Kinglake was not a regular physician, but had "taken out a diploma," I stated the circumstance; but did not charge him with assuming any title which did not belong to him.

This was not done wantonly, or calumniously, as has been asserted; but as an additional argument against placing such implicit confidence in his opinion, as he seemed to expect.

If I accused him of arrogance, and disputed his lofty pretensions, it was not his pretension to a diploma; but to superior judgment, superior medical science, and superior professional skill.

He talks of reprehensive and penal notice, which I may probably receive from another quarter. I have cancelled that part of my work which was founded on erroneous information; and shall be happy to cancel the rest, when he can prove that also to be erroneous, and that his cooling treatment of the gout is not much more to be dreaded than any penal notice for a libel from any quarter under the sun.

He wishes to keep all idea of danger, from his cooling plan, out of contemplation; and, in his allusion to the title of my answer, is cautious not even to mention the word.

You have given him every advantage by noticing what you and he call an unwarrantable assertion, as the first article of Communication; and then setting up a superscription of the same kind: thus shewing a great partiality for your valued correspondent, as you call him, and a fellow-feeling for him. It would be no more than a proof of impartiality, and an act of justice towards me, to prefix a similar expression in both places in your next Journal. We should then see, as the first and most conspicuous article in the Table of Contents, "Mr. Ring, in Answer to an unwarrantable charge;" and, in the first page of the work, "Notice of Dr. Kinglake's unwarrantable Assertion, by Mr. Ring."—We should also see a recantation of the errors into which you have been led by Dr. Kinglake; and of the sarcastic observations artfully brought forward by the conductor, or conductors, of the Journal, by way of preliminary remarks; evidently with the view of prejudicing the public against me, and in favour of Dr. Kinglake and your publication.

*New-street, Hanover-square;*
*December* 10, 1816.

---

**[Our determined adherence to impartiality has induced us to insert the above, but the subject must close here.—EDIT.]**

---

*For the London Medical and Physical Journal.*

